# Timing and pattern of annual silver eel migration in two European watersheds are determined by similar cues

**DOI:** 10.1002/ece3.3099

**Published:** 2017-06-23

**Authors:** Odd Terje Sandlund, Ola H. Diserud, Russell Poole, Knut Bergesen, Mary Dillane, Gerard Rogan, Caroline Durif, Eva B. Thorstad, Leif Asbjørn Vøllestad

**Affiliations:** ^1^ Norwegian Institute for Nature Research (NINA) Trondheim Norway; ^2^ Marine Institute Furnace, Newport Co. Mayo Ireland; ^3^ NINA Research Station Sandnes Norway; ^4^ Institute of Marine Research Storebø Norway; ^5^ Centre for Ecological and Evolutionary Synthesis Department of Biosciences University of Oslo Oslo Norway

**Keywords:** *Anguilla anguilla*, daily variation, environmental variables, freshwater migration, migration onset, silver eel

## Abstract

Many animals perform long‐distance migrations in order to maximize lifetime reproductive success. The European eel migrates several thousand kilometers between their feeding habitats in continental waters (fresh‐, brackish, and sea water) and their spawning area in the Sargasso Sea. Eels residing in freshwaters usually initiate their spawning migration as silver eels during autumn, triggered by diverse environmental cues. We analyzed the time series of silver eel downstream migration in Burrishoole, Ireland (1971–2015), and Imsa, Norway (1975–2015), to examine factors regulating the silver eel migration from freshwater to the sea. The migration season (90% of the run) generally lasted from 1 August to 30 November. Environmental factors acting in the months before migration impacted timing and duration of migration, likely through influencing the internal processes preparing the fish for migration. Once the migration had started, environmental factors impacted the day‐to‐day variation in number of migrants, apparently stimulating migration among those eels ready for migration. Both the day‐to‐day variation in the number of migrants and the onset of migration were described by nearly identical models in the two rivers. Variables explaining day‐to‐day variation were all associated with conditions that may minimize predation risk; number of migrants was reduced under a strong moon and short nights and increased during high and increasing water levels. Presence of other migrants stimulated migration, which further indicates that silver eel migration has evolved to minimize predation risk. The onset of migration was explained mainly by water levels in August. The models for duration of the migration season were less similar between the sites. Thus, the overall migration season seems governed by the need to reach the spawning areas in a synchronized manner, while during the actual seaward migration, antipredator behavior seems of overriding importance.

## 1 INTRODUCTION

**Figure 1:**
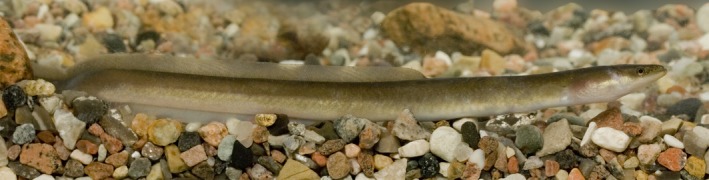
The European eel, *Anguilla anguilla* (L., 1758). Photograph: Nina Jonsson

Migration between different habitats to maximize lifetime reproductive success has evolved in many species within all the major groups of the animal kingdom. Among fishes, the European eel (*Anguilla anguilla,* Figure 1) is an example of a species with a long‐distance migration, covering several thousand kilometers, which is still largely unknown (Aarestrup et al., 2009; Righton et al., 2016). The European eel is panmictic (Als et al., 2011; Palm, Dannewitz, Prestegaard, & Wickstrøm, 2009), and adult fish congregate in the Sargasso Sea to spawn. This is remarkable for such a widespread species (from Mauretania to the Barents Sea), considering the long distance from the continental foraging areas to the spawning area (Tesch, 2003). Timing of migration in the ocean is likely coordinated so that all mature individuals arrive at the spawning areas at the same time.

The abundance of European eel has experienced a serious decline since the 1960s and 1970s (Dekker, 2016). This is well documented in fishery yields as well as in monitoring catches and concerns both silver eels descending and glass eels ascending rivers. Average glass eel recruitment to fisheries in Europe has declined by 97% in the North Sea time series and 89% in other parts of Europe over the last three decades (ICES 2016). The decline has been recorded all over Europe, and there are multiple and partly unknown reasons for this decline. The issue is complex, as negative factors may act in any of the environments inhabited by eels during their life cycle, in freshwater, in inshore waters, as well as in the marine environment—and at all scales, from global warming and associated conditions in the ocean to local hydropower plants in rivers. Thus, among the pressures that might have contributed to the population decline are changes in ocean climate, overexploitation, introduced parasites, pollution, reduced habitat quality and quantity, and mortality during migration (e.g., Calles et al., 2010; Castonguay & Durif, 2015; Durif, Gjøsæter, & Vøllestad, 2011; Geeraerts & Belpaire, 2010; Lefebvre, Fazio, Mounaix, & Crivelli, 2013). To promote recovery of the stock, a reduction in mortality is required. This article contributes to identify and possibly predict peak periods for silver eel migration, facilitating targeted protection measures, particularly in relation to water flow regulation and operation of hydropower facilities. Our conclusions are therefore highly policy relevant.

Environmental factors may influence the preparation of individual animals to migrate, and serve as cues to initiate the migration. In European eel, the metamorphosis from yellow to silver eels before the marine migration to the spawning area in the Sargasso Sea includes morphological, anatomical, as well as physiological changes and occurs during summer (Durif, Dufour, & Elie, 2005; van Ginneken et al., 2007). The subsequent downstream migration to sea for the freshwater‐living component of the eel mainly occurs during autumn (Deelder, 1984; Vøllestad et al., 1986). In some cases, a substantial proportion of the migration may also occur in spring (Aarestrup et al., 2008; Reckordt, Ubl, Wagner, Frankowski, & Dorow, 2014; Stein et al., 2016). One of the strategies employed by animals to coordinate migration and reproduction is by responding to variations in large‐scale environmental factors. Thus, the onset of the silver eel migration may be influenced by a number of factors, such as day length, light conditions during the dark hours (i.e., moon phase), water level and water temperature (Durif & Elie, 2008; Vøllestad, Jonsson, Hvidsten, & Næsje, 1994). These factors may interact. For instance, Vøllestad et al. (1986) demonstrated earlier migration after a cool than after a warm summer and that increased water levels triggered migration. Change in water level may also trigger changes in migration activity in different ways according to the time of the season (Vøllestad et al., 1986). The relationship between environmental factors and the onset of the downstream silver eel migration period is complex and may vary among rivers and years. Long‐term data series are needed to develop models that can be used to understand this relationship, an understanding that can be used to predict peak migration and reduce mortality at barriers such as those for hydropower.

In this study of more than 40 years, we aimed to understand the relationship between environmental factors and migrating silver eels, by analyzing the two unique long‐term data series of eel migration from the Burrishoole catchment in Ireland and the River Imsa catchment in Norway, where all out‐migrating silver eels have been captured in traps close to the sea and registered daily since 1971 and 1975, respectively. These data are fisheries independent and not constrained by fishing regulations. This enabled us to identify migration cues and compare the impacts of these cues in two different geographic areas with different climatic conditions. We analyzed the effects of environmental factors in the months before as well as during the migration, to identify factors that may impact the preparations to become ready to migrate, as well as cues initiating the migration.

## 2 MATERIAL AND METHODS

**Figure 2:**
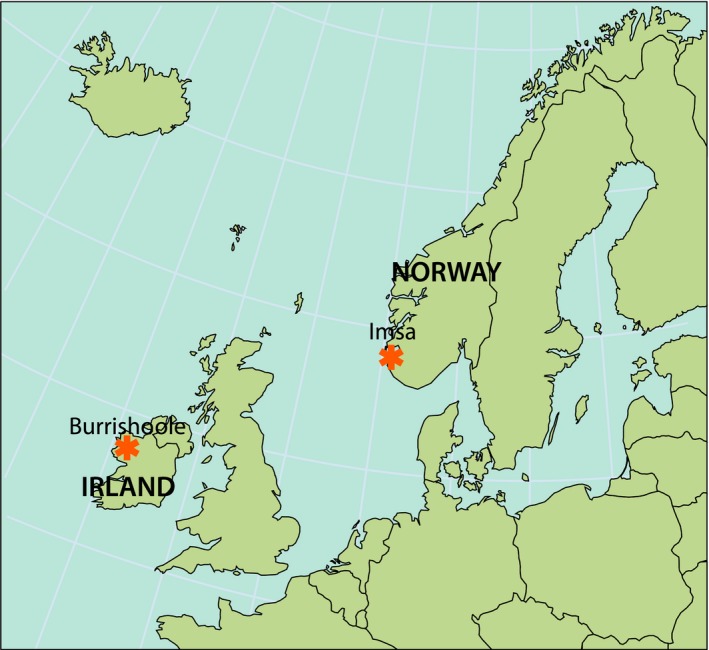
Map of northwestern Europe, showing the location of the two study sites (stars), which are Burrishoole in Ireland (53.94889°N 9.57556°W) and Imsa in Norway (58.9037°N 5.96428°E). Detailed maps of the two watercourses are in [Link], [Link]. Map source: MAP Art/NINA

The Burrishoole catchment, Co. Mayo, western Ireland (Figure 2 and [Link], [Link]) is an oligotrophic and poorly buffered system. The catchment has an area of 8,949 ha, of which 450 ha (5.0%) is lake surface (lakes Feeagh 395 ha, Bunaveela 46 ha and a number of smaller lakes). Upstream and downstream Wolf‐type fish traps, employing horizontal grids with 10‐mm gaps, are situated on two short outflow rivers (Mill Race and Salmon Leap) from Lake Feeagh to the brackish Lake Furnace. The distance from the traps to the upper end of eel habitat in the Burrishoole catchment is approx. 13 km. Trapping commenced on the Mill Race in 1958 and full trapping of all migrating silver eels commenced at both outflows in 1971 (Poole, Reynolds, & Moriarty, 1990). Water level in Lake Feeagh is taken as a proxy for water flow at the Wolf traps which are situated circa 50‐m downstream from the lake.

The River Imsa, in the Rogaland County, southwestern Norway (Figure 2 and [Link], [Link]), is also an oligotrophic system. The catchment covers an area of 12,800 ha, of which 1,536 ha (12%) is lake surface (major lakes are Imsvatnet, 40 ha, and Storavatnet, 819 ha). All descending fish are caught in a Wolf trap (apertures 10 mm, inclination 1: 10) situated about 100 m from the river outlet in the sea. The distance from the trap to the upper end of eel habitat in the Imsa catchment is approx. 20 km, and the distance from the nearest lake (Imsvatnet) along the free‐flowing river to the fish trap is approx. 970 m. The trap has been in operation throughout the year since 1975. Water discharge is estimated based on an empirical relationship between river water level and discharge, and the discharge has varied between 0.02 and 34 m^3^/s, with an annual mean of 5.1 m^3^/s, over the recorded period. Hereafter, “water level” is used to denote water flow through the Wolf traps in both Burrishoole and Imsa.

Wolf traps with aperture 10 mm generally catch all eels larger than approx. 25 cm in length (Vøllestad & Jonsson, 1986). This would include virtually all silver eels in Burrishoole and Imsa (Poole et al., unpublished data). The data used in this analysis are the daily number of silver eels caught in the Wolf traps. At both Burrishoole and Imsa, the traps were checked twice every day (at circa 08:00 and 15:00 h) throughout the year. Water level and water temperatures were recorded daily. Water flow is unregulated in both rivers. In both rivers, there was a shift in annual number of migrating silver eels in the 1980s (Poole et al., unpublished data). In Burrishoole, the shift occurred in 1982, with the mean number shifting from 4,445 to 2,765 eels. In Imsa, the shift in mean numbers occurred in 1988, from 5,815 to 2,201 eels. There has been no stocking of eels in these water courses, so all silver eel production is based on natural glass eel recruitment.

Over the study period, mean annual water temperatures in Burrishoole varied between 9.2 and 12.4°C (total mean 10.5°C) and in Imsa between 7.7 and 10.7°C (total mean 9.4°C). The same difference of 1.1 degrees was seen in the recorded mean water temperature during the migration season (1 August–30 November), with 13.3°C at Burrishoole and 12.2°C at Imsa. An examination of the temperature anomalies compared to the period 1971–2000 (1975 for Imsa) indicated a consistent period of warming from 1997/1998 to 2015 (Fealy et al., 2014; Poole et al., in prep.).

### 2.1 Statistics and modeling

The statistical analyses and modeling were carried out using the statistical software R (R Core Team 2017, v. 3.3.3). The daily catches of migrating eels were modeled by generalized linear models (GLMs), with eel counts assumed to be Poisson distributed (Dalgaard, 2008). In a GLM with a Poisson distributed response, it is important to check for overdispersion, that is, whether the error distribution has a variance larger than expected from the model. To account for overdispersion and obtain a more appropriate variance function, a quasi‐Poisson likelihood was used (Crawley, 2007). For day number *x*, the number of remaining eels ready for migration was estimated as the difference between the total number of migrants recorded during the season minus the number of migrants recorded up to and including day *x−1*. The number of remaining eels was used as an offset term in the model. For a model with a quasi‐Poisson likelihood, the AIC (Sakamoto, Ishiguro, & Kitagawa, 1986) is not defined, but model simplification from the maximum model, which includes all explanatory variables, can be performed using the deviance to test between different model alternatives (*F* test, Dalgaard, 2008). Due to the large sample size of daily eel catches, several coefficients became significant although barely improving the explanatory power of the models. A subsequent model simplification was therefore repeated until we obtained a more parsimonious model. The model was fitted without the least significant variable and the *R* ^2^ calculated. If we observed no major reduction in *R* ^2^ (reduction in *R* ^2^ not greater than .005), the variable was omitted.

For the annual onset, and duration, of migration models, generalized linear regression models were fitted. A maximum model including all relevant explanatory variables was simplified by stepwise reduction based on the AIC. If consecutive monthly averages for either water level or temperature had the same effect on the response; that is, the estimates had the same sign, a model where these months were pooled together was also fitted and evaluated. For example, the mean water temperatures for June, July, and August could be pooled together to give the mean summer temperature. A decrease in AIC of more than two was considered as sufficient support for retaining a variable in the model. Residuals were checked for normality and autocorrelation.

### 2.2 Model parameters

#### 2.2.1 Response variables

We defined the migration season as lasting from 1 August to 30 November. In both rivers, the mean proportion of eels migrating during this period was 96.7%. There was no peak in migration in spring, as the mean proportion of eels leaving the rivers during March–May was 0.2% and 0.3% in Burrishoole and Imsa, respectively. The corresponding proportion of fish migrating during winter (December–April) was 1.3% and 1.4%. We developed models for three response variables (Table 1). These were the annual onset of migration (D5), the duration of migration (D5‐D95), and daily number of migrants (N_day_). We defined the onset of migration as the day in the season when 5% of the eels have migrated and duration as the number of days between D5 and D95, that is, the period when 90% of the eels migrated. Thus, we considered the few fish that migrated before D5 and after D95 as aberrant migrants and of little consequence for the annual silver eel migration. However, it should be noted that in years with few migrating eels, a few aberrant fish may have a large impact on D5. The day when 50% of the season's migrants had been recorded (D50) was also investigated, but no significant model was found for this response variable, so no results are presented.

**Table 1:** Responsive and explanatory variables included in models for silver eel migration at Burrishoole and Imsa

Variable name	Meaning
Response variables	
N_day_	Number of eels in trap per day
D5	Onset of migration; Julian day number when 5% of the season's total number of eels had migrated
D50	Julian day number when 50% of the season's total number of eels had migrated
D95	Julian day number when 95% of the season's total number of eels had migrated
D95‐D5	Duration of the migration season. The number of days from D5 to the day when 95% of the season's total number of eels had migrated
Explanatory variables—annual models	
WL_’month’_	Water level denotes standardized mean water flow through the Wolf traps in month (subscript) for all months from December the previous year until and including November this year. In Burrishoole, Lake Feeagh water level is used as a proxy and in Imsa river discharge is measured directly
Year	Year of sampling
T_‘month’_	Mean water temperature in month (subscript) for all months from December the previous year until November this year
Explanatory variables—daily model	
δW**L**	Water level change from the day before. Change over the last 3, 5, and 7 days was tested and rejected
T	Water temperature (recorded at midnight in Burrishoole, at 8 AM in Imsa)
T_week_	Mean water temperature during the preceding week
T_devX_	Daily temperature deviation (absolute value) from optimum temperature (X) for migration. Based on our own observations, the approximate values of X were 9°C in Imsa and 11°C in Burrishoole
WL	Daily water level (standardized)
N_day‐1_	Number of eels in trap the previous day
Moon	Continuous index between 0 (no moon) and 1 (full moon), indicating the proportion of the moon being illuminated. Also in daily model
ND_rem_	Days remaining of the migration season – migration season set to start 1 August and end 30 November
Offset variables	
N_rem_	Number of migrants remaining upstream of trap

For Imsa, a few of the daily observations were zero catch 1 day and large catches the days before and after. As the zero catch was caused by the trap not being emptied, these were adjusted by splitting the catch from the following day in two, with one half assigned to the day with zero catch.

#### 2.2.2 Explanatory variables

All explanatory variables were associated with variation in the environment, except the number of migrants the previous day (N_day‐1_, Table 1). Three environmental factors that may affect silver eel migration were included, which were water level, water temperature, and moon phase. When we are fitting multivariate models from a set of environmental variables, we will never have completely independent explanatory variables. One must be cautious when interpreting significant coefficients of the model; no single term can be interpreted independently of the others. In this case, this consideration is particularly valid for water level and water temperature. In both Burrishoole and Imsa, there was a negative correlation between water level and water temperature in each of the summer months May–August; that is, high waters were associated with low temperatures and vice versa.

The onset of migration is an annual observation, which requires explanatory variables (water level, temperature) that are averaged or accumulated over some time period, associated with fixed dates or periods before the onset of migration. Moon phases were represented by a continuous variable indicating the visible proportion of the moon between 0 (no moon) and 1 (full moon).

The number of eels migrating on any particular day may be influenced by events before that day, and therefore, water level, water temperature, light conditions, and the number of eels that migrated the previous day were included in the model. It was assumed that the total number of eels caught in the trap through a season represents the annual total migrating stock so the number of fish still to migrate within the season was used as model offset. This implies that the estimated number of migratory eels per day depends on the available number of silver eels remaining upstream of the trap. The number of days remaining until the end of the migration season was also used as an explanatory variable in the model, with the number of dark hours increasing until the end of the season. To use this variable in the predictive model, the end of annual migration was set to 30 November, as very few fish usually were recorded after this date.

In order to develop a common model for the two rivers, some modifications of the variables were required. The water level time series was standardized, because we assumed that it is not the absolute water level that is informative, but rather the variation around the mean, and the rate of change over time. Whether we used the standardized or the original variable did not affect the performance of the two separate models, but the estimated parameters changed. Using the standardized variables in the common model, we assumed that the variation around the mean had similar effect.

## 3 RESULTS

### 3.1 Timing of the silver eel migration

**Figure 3:**
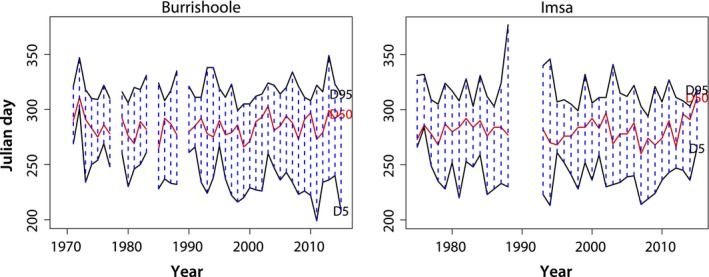
The timing (Julian day number) of silver eel migration in Burrishoole (1971–2015) and Imsa (1975–2015) described by the onset (D5) and end (D95) of migration, as well as when 50% (D50) of the season's eels had been recorded

The pattern of silver eel migration, including the timing of migration onset (D5) and end (D95), varied greatly among years both in Burrishoole and in Imsa. The start of the annual migration (D5) varied from 18 July to 28 October in Burrishoole and from 1 August to 11 October in Imsa (Figure 3). Associated with this variation in D5 is substantial variation in water level and temperature. In Burrishoole, water level in Lake Feeagh at D5 has been between 0.16 and 0.85. In Imsa, river discharge at D5 has varied between 0.26 and 12.78 m^3^/s. Corresponding values for temperatures were between 12.0 and 17.8°C for Burrishoole and between 10.6 and 22.1°C for Imsa. In Burrishoole, the onset of migration exhibited a temporal trend as the migration season started on average 0.8 days earlier per year over the sampling period. There was no such significant trend in the Imsa material, where the mean date for the onset of silver eel migration was 28 August. There were no discernible temporal trends in D50 or D95 in either catchment. The end of migration (D95) occurred between 26 October and 15 December in Burrishoole and between 21 October and 12 January in Imsa. However, in both watersheds, D95 rarely occurred after 1 December. Interestingly, the date when half the fish had migrated (D50) varied within an almost identical time period in the two rivers: 21 September–7 November in Burrishoole and 17 September–7 November in Imsa.

**Figure 4:**
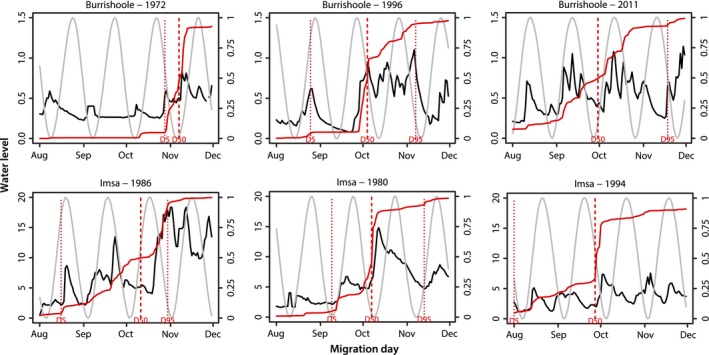
The pattern of seasonal silver eel migration (cumulative curve for numbers of eel migrating, red), water level (black line: m^3^/s), and proportion of moon showing (gray line: 0; no moon, to 1; full moon), for selected years in Burrishoole and Imsa. The onset (D5), 50% (D50), and end (D95) of migration season are indicated for each year

A late onset of migration often led to a burst of migration with 50% (D50) of the migrants recorded after a few days (Figure 4) and usually resulting in a short migration season (cf. Figure 3). It was noticeable that in both rivers, an early start of migration resulted in a long migration season.

### 3.2 Daily fish counts

As the datasets for daily fish counts were large, the model preferred by the deviance test tended to include most explanatory variables as significant or nearly significant. However, removing all the insignificant variables, as well as those that were significant but had little additional effect on model performance (as assessed by the change in *R* ^2^), left us with five explanatory variables for both Burrishoole and Imsa (Table 2A and C), of which four were common for both watersheds (Table 2B and D). In both watersheds, standardized daily water level and the number of migrants caught the day before had a positive impact on the number of migrants, while moon illumination and number of days remaining in the migration season had a negative impact. In Burrishoole, this model explained 56% of the variation (Table 2B), down from 58% for the maximum model (not shown). Adding the fifth significant variable, which was water level change (with a positive impact on the number of migrants, Table 2A), had only a marginal positive effect on the model (*R* ^2^ = .561). The preferred model for daily number of eels in Imsa including the four explanatory variables similar to Burrishoole explained 56% of the variation (Table 2D), compared to 57% for the maximum model (not shown). The fifth significant variable at Imsa, which was deviation from the optimal temperature, had only a marginal effect on model performance (*R* ^2^ = .566; Table 2D).

**Table 2:** Model parameters for predicting the number of migrating silver eels per day (N_day_) for Burrishoole (A and B) and Imsa (C and D). For explanation of variables, see Table 1

	Coefficients:	Estimate	*SE*	*t* Value	Pr(>|*t*|)
A	Burrishoole				
	(Intercept)	−3.098	0.069	−45.01	<2e‐16
	WL	0.496	0.017	29.75	<2e‐16
	Moon	−0.689	0.059	−11.64	<2e‐16
	ND_rem_	−0.032	0.001	−41.20	<2e‐16
	ln(N_day‐1_)	0.354	0.011	32.55	<2e‐16
	δWL	0.270	0.011	24.46	<2e‐16
B	Burrishoole				
	(Intercept)	−3.051	0.071	−42.88	<2e‐16
	WL	0.560	0.018	31.97	<2e‐16
	Moon	−0.726	0.062	−11.79	<2e‐16
	ND_rem_	−0.031	0.001	−39.11	<2e‐16
	ln(N_day‐1_)	0.337	0.011	29.85	<2e‐16
C	Imsa				
	(Intercept)	−3.350	0.087	−38.71	<2e‐16
	WL	0.237	0.022	10.69	<2e‐16
	Moon	−0.584	0.067	−8.66	<2e‐16
	ND_rem_	−0.027	0.001	−18.89	<2e‐16
	ln(N_day‐1_)	0.466	0.016	−3.42	<2e‐16
	T_dev9_	−0.052	0.015	29.58	0.001
D	Imsa				
	(Intercept)	−3.378	0.086	−39.50	<2e‐16
	WL	0.235	0.022	10.50	<2e‐16
	Moon	−0.565	0.068	−8.29	<2e‐16
	ND_rem_	−0.030	0.001	−32.53	<2e‐16
	ln(N_day‐1_)	0.482	0.015	32.47	<2e‐16

As both selected models (Burrishoole Table 2B, Imsa Table 2D) included the same four explanatory variables contributing in the same direction, we used cross‐validation between rivers to test their prediction efficiency. Predicting Imsa daily catches using the Burrishoole model gave an *R* ^2^ = .53, instead of *R* ^2^ = .56 for the Imsa model. Conversely, predicting the Burrishoole daily catches from the Imsa model resulted in *R* ^2^ = .52, a decrease of only 0.04 compared to the Burrishoole model.

A common model for daily counts of fish in the traps for both rivers was almost as good as the separate models (*R* ^2^ = .54, Table 3). In addition to the four explanatory variables that were common for the two separate models, relative water level change made a significant and positive contribution in the common model.

**Table 3:** Model parameters in a common model for Burrishoole and Imsa for predicting the number of migrating silver eels per day (N_day_). For explanation of variables, see Table 1

Coefficients	Estimate	*SE*	*t* Value	Pr(>|*t*|)
(Intercept)	−3.118	0.053	−58.48	<2e‐16
WL	0.390	0.014	28.73	<2e‐16
δWL	0.266	0.009	28.06	<2e‐16
Moon	−0.678	0.045	−15.04	<2e‐16
ln(N_day‐1_)	0.383	0.009	43.40	<2e‐16
ND_rem_	−0.032	0.001	−52.48	<2e‐16

### 3.3 Onset of migration

#### 3.3.1 Burrishoole

The model describing the onset of silver eel migration in Burrishoole included year and standardized mean water level in August as significant explanatory variables (AIC = 346.3, down from 357.3 for the maximum model) and explained 49% of the variation in D5. Year was included in this model because the time series plot indicated that migration started earlier (i.e., a decreasing trend in D5) over time (cf. Figure 3). The unexplained variation was partly due to some few aberrant individual years, for example, 1972, when D5 was very late due to a prolonged drought (cf. Figure 4). To examine the role of other variables with a potential covariance with year, year was removed from the model, resulting in a maximum model including all explanatory variables with AIC = 367.5 (*R* ^2^ = .62). After a reduction of the number of variables, an improved model with AIC = 361.7 was selected (*R* ^2^ = .30; Table 4A). The water level in August was retained as the most significant variable, with high water level causing early D5. High water temperature in May also contributed to an early D5, while high mean temperature in July had the opposite effect, that is, a late D5. The impact of the temperature variables was not significant (*p* > .05). However, a model including only the water level in August as explanatory variable (Table 4B) only explained 22% of the variation, even if the AIC barely increased (AIC=362.1). The water temperatures in May and July were therefore retained to facilitate the comparison with Imsa.

**Table 4:** Model parameters for predicting the onset of silver eel migration in Burrishoole (A and B) and Imsa (C and D). For explanation of variables, see Table 1

	Model	AIC	Coefficients	Estimate	*SE*	*t* Value	Pr(>|*t*|)
A	Burrishoole	361.7	(Intercept)	104.5	36.284	2.879	0.0065
			T_May_	−3.98	2.078	−1.916	0.063
			T_Jul_	3.45	2.396	1.439	0.16
			WL_Aug_	−13.50	4.924	−2.742	0.0093
B	Burrishoole	362.1	(Intercept)	115.45	2.97	38.82	<2e‐16
			WL_Aug_	−15.85	4.74	−3.346	0.00179
C	Imsa	291.6	(Intercept)	97.52	27.06	3.60	0.001
			T_May_	−2.80	1.49	−1.88	0.07
			T_Jul_	2.48	1.45	1.71	0.096
			WL_Aug_	−19.25	5.04	−3.82	0.001
D	Imsa	291.7	(Intercept)	108.77	3.45	31.50	<2e‐16
			WL_Aug_	−21.44	5.16	−4.16	0.0002

#### 3.3.2 Imsa compared to Burrishoole

The maximum model for D5 at Imsa with all explanatory variables included had an AIC of 296.1 (*R* ^2^ = .74). The reduced model with the three same variables as at Burrishoole explained 42% of the variance in D5 (AIC=291.6; Table 4C). Also in Imsa, high mean water temperatures in May and high water level in August caused an earlier D5, while a warm July caused a delay in D5. Explanatory variables for the two rivers were the same, with the same sign, and with quite similar coefficient values. As at Burrishoole, the temperature variables were not significant in Imsa (*p* > .05). A model including only water level in August as explanatory variable had a slightly higher AIC, but a much lower explanatory power (33%).

As the selected models for onset of migration (D5) in Burrishoole (Table 4A) and Imsa (Table 4C) contained the same explanatory variables contributing in the same direction, a cross‐validation between rivers was used to test prediction efficiency. Predicting Imsa D5 using the Burrishoole model (cf. Table 4A) gave *R* ^2^ = .38, instead of *R* ^2^ = .42 for the Imsa model (Table 4C). Conversely, applying the Burrishoole data to the Imsa model (cf. Table 4C) resulted in *R* ^2^ = .30, which was similar to the value obtained in the original model (Table 4A). However, there was a major difference in the models. When the Burrishoole model was applied to the Imsa data, D5 was predicted on average 8 days later than observed, while the Imsa model applied to the Burrishoole data predicted D5 on average 8 days earlier than observed.

### 3.4 Duration of the migration season

#### 3.4.1 Burrishoole

A model for the duration of the migration season (D95‐D5) in Burrishoole included year and six explanatory variables, of which four were associated with temperature and two with water level, had a high explanatory power (*R* ^2^ = .79; AIC = 319.1, compared to an AIC of 322.9 for the maximum model). High February and November temperatures contributed to a longer migration period, whereas high temperatures in December the previous year and in August caused a shorter duration. High water level in August caused a longer migration period, while high water level in November resulted in a shorter migration period. Removing year from the model while keeping the six temperature and water level variables resulted in *R* ^2^ = .60 and AIC = 343.8.

If the water level variables were removed from this model, the simplified model explained 53% (Table 5; AIC=346.17). The temperatures retained their impact on the duration of migration, with high temperatures in December the previous year and in August causing a reduced migration period, and water temperatures in February (previous) and November causing a longer period. No significant interactions were found between the temperatures.

**Table 5:** Model for the duration of silver eel migration in Burrishoole with water temperatures as explanatory variables. For explanation of variables, see Table 1

Coefficients	Estimate	*SE*	*t* Value	Pr(>|*t*|)
(Intercept)	144.72	35.20	4.11	0.0002
T_Dec‐1_	−8.91	2.73	−3.27	0.002
T_Feb_	7.98	2.27	3.51	0.001
T_Aug_	−7.86	1.77	−4.45	7.7e‐05
T_Nov_	9.01	2.21	4.09	0.0002

#### 3.4.2 Imsa

The maximum model for the duration of the migration season (D95‐D5) in Imsa explained 86% of the variation (AIC=311.7). A reduced model including three temperature variables (previous December, February, March) and two water level variables (August and November) also performed well, explaining 40% of the variation (Table 6; AIC = 319.8). In this model, high temperatures in previous December and March and high water level in November contributed to a shorter migration season. High temperature in February and high water level in August had the opposite effect.

**Table 6:** Model parameters for predicting the duration (D95‐D5) of silver eel migration in Imsa. For explanation of variables, see Table 1

Coefficients	Estimate	*SE*	*t* Value	Pr(>|*t*|)
(Intercept)	133.48	17.41	7.67	1.5e‐08
T_Dec‐1_	−5.94	3.36	−1.77	0.09
T_Feb_	13.85	7.36	1.88	0.07
T_Mar_	−15.18	6.85	−2.22	0.03
WL_Aug_	22.93	7.57	3.03	0.005
WL_Nov_	−7.09	3.95	−1.79	0.08

#### 3.4.3 Comparison between Burrishoole and Imsa

The monthly temperatures included in the selected models for the duration of migration from both rivers, that is, previous December and February, acted in the same direction in both cases. In both rivers, water level in August and November also acted in the same direction.

A cross‐validation of the two models for duration of the migration season, by applying the Burrishoole data to the Imsa model, and vice versa, was less successful than cross‐validation of the models concerning daily number of migrants and onset of migration. Applying the Imsa data to the Burrishoole model based on six explanatory variables (two associated with water level and four associated with temperature) resulted in low explanatory power; 11% compared to 40% for the Imsa model. Applying the Imsa data for the duration of migration to the Burrishoole model, which included both water level and temperature variables (cf. Table 6), also resulted in substantially lower explanatory power; 26% compared to 60%.

## 4 DISCUSSION

The annual timing, duration, and pattern of silver eel migration in the rivers Burrishoole and Imsa exhibited great variation, but migration nearly always occurred within a restricted period (1 August–30 November). The seasonality of silver eel migration in these rivers is in accordance with the general observations in Europe; downriver migration occurs in autumn and early winter (Deelder, 1984; Tesch, 2003). Migration occurs earlier in the year in the north (Bergersen & Klemetsen, 1988; Davidsen et al., 2011; Huitfeldt‐Kaas, 1904), which may be associated with lower temperatures.

The impact of environmental variables on the annual timing, duration, and pattern of silver eel migration indicated that the environment may act along different time scales and apparently on different aspects of migration. Environmental factors acting in the months before migration had an impact on the onset and duration of migration. Temperatures during spring and summer likely influence the physiological, morphological and energetic preparations for migration. Environmental factors like water temperature and food availability probably act in concert to promote accumulation of muscular fat, which is an important factor preparing the fish for migration (e.g., Belpaire et al., 2009). Significant impact of water temperatures, already from the previous December in both rivers, indicates that the environment may influence physiological processes related to silvering of the fish over an extended period, although silvering itself is believed to commence in spring, following a peak in the production of growth hormones (Durif, Van Ginneken, Dufour, Müller, & Elie, 2009; Durif et al., 2005). Favorable growth conditions enhance the silvering process (Durif et al., 2005), and therefore, food availability and water temperatures will determine how fast the silvering occurs. This is similar to smoltification in anadromous salmonids, which is also a process lasting over an extended time period (e.g., Jonsson & Jonsson, 2011). In northern inland waters, productivity and temperatures are low. Spring comes late and water temperatures in some cases rarely exceed 10–12°C. Because silver eels in northern areas migrate early (Bergersen & Klemetsen, 1988; Davidsen et al., 2011), it seems likely that the internal processes associated with silvering needs to last longer than over a few summer months. Due to a shorter period available for growth at these latitudes, temperature variation likely has more direct consequences.

When the silvering process is completed, environmental conditions act on a shorter time scale. In particular, water levels appear to be important, as high water levels in August hasten the onset of migration, while low water levels or drought conditions may impede migration. This may be a particular problem in small catchments such as Burrishoole and Imsa, where water discharge responds quickly to changes in precipitation. As low waters are associated with high temperatures, the impact on migration of these two variables cannot be evaluated independently. Once the migration had started, environmental factors impacted the day to day variation in numbers of migrants, apparently serving as cues to stimulate migration among those eels that were ready for migration.

On the short time scale, the daily number of migrants was positively correlated to high water levels and the number of migrants the day before, while strong moonlight and being early in the season had a negative impact. A positive relationship between water level and the number of migrating silver eels is a common observation (Deelder, 1984; Frost, 1950; Haro, 2003; Trancart, Acou, De Oliveira, & Feunteun, 2013). Migrating with high waters is interpreted partly as an antipredator behavior, but it is also energetically beneficial to migrate at higher water levels when water velocity is higher (Barry et al., 2016).

A high number of migrants the day before led to a high number of migrants indicating that eels may be triggered to migrate by other conspecifics moving. Such direct stimulation to migrate by social cues has not, to our knowledge, been shown in previous studies of silver eel migration. However, silver eels migrate in groups (Bruijs & Durif, 2009), and in Lough Derg, in Ireland, they aggregate before migrating as a group when favorable conditions emerge (McGrath, O’ Leary, Sharkey, & Murphy, 1979). Moving in large numbers also provides protection against predation.

Silver eels are known to migrate in dark and moonless nights (Frost, 1950; Lowe, 1952). Our observations of a negative impact of strong moonlight and short nights (i.e., early in the season) on the number of migrating eels are in line with this statement and with a number of other studies (Breukelaar et al., 2009; Cullen & McCarthy, 2003; Haraldstad, Vøllestad, & Jonsson, 1985; Vøllestad et al., 1986). The silver eels tend to migrate during the last quarter moon (Tesch, 2003), but other studies indicate that moon phase is less important and that the negative effect of moonlight can be obscured by cloud cover or turbid water which cause low visibility (Bruijs & Durif, 2009; Cullen & McCarthy, 2003; Reckordt et al., 2014). This indicates that the observed pattern of reduced movement in strong moonlight is related to light conditions rather than moon phase per se and that eels prefer to migrate in the dark. This is likely also an antipredator behavior.

The models describing the daily number of migrating eels in Burrishoole and Imsa were almost identical, with similar parameter values. The common model, which included the relative increase in water level as a fifth explanatory variable, also had high explanatory power. Moreover, all explanatory variables that were important in the models explaining the daily number of migrants are likely associated with antipredation behavior.

The temporal trend of earlier onset of migration (D5) in Burrishoole was not observed in Imsa, in spite of a similar general increase in annual mean temperature at both localities (Poole et al., unpublished data). In Burrishoole, the onset of migration (D5) became on average 0.8 days earlier per year, implying that D5 was 35 days earlier in 2015 than in 1971. A completed silvering process is a precondition for migration. Silvering is triggered by growth (Durif et al., 2005; Huang et al., 1998), and the onset of migration may therefore be correlated with when the yellow eels commence growing in spring. Eels remain dormant under low temperatures in winter and become active when temperatures rise in spring. The minimum temperature for activity (and presumably feeding) seems to vary among sites. In Lake Mälaren, Sweden, Westerberg and Sjöberg (2014) recorded that eel activity in spring commenced at temperatures above 3–7°C. In Imsa, Haraldstad et al. (1985) caught feeding yellow eels at 8°C, and in northern Norway, activity has been recorded at even lower temperatures (Bergersen & Klemetsen, 1988). In a small Spanish stream, Costa‐Dias and Lobon‐Cervia (2008) recorded some feeding activity even at the minimum winter temperatures of around 6°C, but with increased feeding rates in April–May, when temperatures reached 9–10°C. Although experiments may indicate that somatic growth only commences at higher temperatures, above 13–15°C (Tesch, 2003), fish under natural conditions and acclimatized to lower temperatures may differ (cf. Walsh, Foster, & Moon, 1983). Thus, the temperature when eels start feeding and growing in spring may depend on acclimatization to local conditions, and the onset of growth may be governed on a finer scale by water temperature rise in spring. It may be speculated that the trigger temperature for growth in spring has been reached earlier over the last forty years in Burrishoole, while this has not been the case in Imsa. However, this issue cannot be resolved with the data available to us.

Removing year as an explanatory variable from the Burrishoole model for the onset of migration resulted in almost identical models for the onset of migration in the two rivers. High water temperatures in May contributed to an early onset of the annual migration, while high water temperatures in July tended to delay migration onset. Thus, a warm spring seems to cause an early migration, whereas a warm summer delays migration (Cullen & McCarthy, 2003; Durif & Elie, 2008). It may be speculated that a warm May enhances the early physiological processes in the fish associated with silvering, preparing it for an early migration. In these rivers, a warm July is associated with low water levels, which will delay the fish that are ready for migration. The fact that high mean water level in August caused an early onset of migration indicates that the migration‐ready silver eels utilize the favorable water levels as soon as they occur. The impact of water levels in August is in accordance with the earlier observations by Vøllestad et al. (1986) in Imsa and is also valid for Burrishoole.

Applying the model for the onset of migration (D5) from one river to the data from the other river confirmed the similarity of the systems, except for the fact that in this case, the predicted D5 differed by, on average, 8 days from the observed D5. The Burrishoole model applied to Imsa data resulted in a delay of predicted D5 compared to the observed D5 in Imsa. In the reverse situation, applying the Imsa model to the Burrishoole data leads to the predicted D5 being 8 days earlier that the observed D5 in Burrishoole. One possible reason for this may be related to the position of the Wolf traps. While the trap in Burrishoole is situated immediately below the lake, there is a river stretch of nearly 1 km from the lowermost lake to the trap in Imsa. Moreover, there are several relatively large lakes with eels upstream in the Imsa watershed. Thus, the Imsa eels may spend longer time than the Burrishoole eels, from initiating their migration in the lakes until they reach the trap.

The only explanatory variables in common for the models to explain variation in the duration of the migration in the two rivers were water temperature in December the previous year, as a warm December seems to contribute to a short migration season, and a warm February, which causes a longer season. The mechanisms behind these observations are not known, and any explanation remains speculative. However, it may indicate that the internal processes ending with the silvering of the eels may be more extended in time than previously reported.

In conclusion, the data series on silver eel migration in Burrishoole and Imsa show that the daily number of migrating eels and the onset of migration are influenced by the same explanatory variables in the two rivers. It may appear that the day‐to‐day variation in number of migrating eels mainly is governed by variables which combine to provide the best conditions to avoid predators. The need to reach the spawning areas in the Sargasso Sea at a certain time of the year may play a lesser role on this restricted time scale. The general timing of migration in the autumn, however, may be associated with the need to congregate in the spawning areas at a certain time. Still, the same environmental variables explain the onset of migration in both rivers, in spite of Imsa being more than 1000 km further away from the Sargasso Sea than Burrishoole. The models explaining the duration of the migration season differed more between the two rivers, but in both cases, water temperatures over the preceding year and water levels were important variables.

## CONFLICT OF INTEREST

None declared.

## AUTHOR CONTRIBUTIONS

Odd Terje Sandlund involved in conception and design, interpretation of data, lead author, drafting, and revising. Ola H. Diserud involved in conception and design, analysis of data, and modeling, drafting, revising. Russell Poole involved in conception and design, acquisition of data, maintenance of data series, interpretation of data, and revising. Knut Bergesen involved in acquisition of data, maintenance of data series, and revising. Mary Dillane involved in acquisition of data, maintenance of data series, and revising. Gerard Rogan involved in acquisition of data, maintenance of data series, and revising. Caroline Durif involved in conception and design, interpretation of data, and revising. Eva B. Thorstad involved in conception and design, interpretation of data, drafting, and revising. L. Asbjørn Vøllestad involved in conception and design, interpretation of data, and revising.

## References

[ece33099-bib-0001] Aarestrup, K. , Økland, F. , Hansen, M. M. , Righton, D. , Gargan, P. , Castonguay, M. , … McKinley, R. S. (2009). Oceanic spawning migration of the European eel (*Anguilla anguilla*). Science, 325, 1660. 19779192 10.1126/science.1178120

[ece33099-bib-0002] Aarestrup, K. , Thorstad, E. B. , Koed, A. , Jepsen, N. , Svendsen, J. C. , Pedersen, M. I. , … Økland, F. (2008). Survival and behaviour of European silver eel in late freshwater and early marine phase during spring migration. Fisheries Management and Ecology, 15, 435–440.

[ece33099-bib-0003] Als, T. D. , Hansen, M. M. , Castonguay, M. , Riemann, L. , Aarestrup, K. , Munk, P. , … Bernatchez, L. (2011). All roads lead to home: Panmixia of European eel in the Sargasso Sea. Molecular Ecology, 20, 1333–1346. 21299662 10.1111/j.1365-294X.2011.05011.x

[ece33099-bib-0004] Barry, J. , Newton, M. , Dodd, J. A. , Lucas, M. C. , Boylan, P. , & Adams, C. E. (2016). Freshwater and coastal migration patterns in the silver‐stage eel *Anguilla anguilla* . Journal of Fish Biology, 88, 676–689. 26707686 10.1111/jfb.12865

[ece33099-bib-0005] Belpaire, C. G. J. , Goemans, G. , Geeraerts, C. , Quataert, P. , Parmentier, K. , Hagel, P. , & De Boer, J. (2009). Decreasing eel stocks: Survival of the fattest? Ecology of Freshwater Fish, 18, 197–214.

[ece33099-bib-0006] Bergersen, R. , & Klemetsen, A. (1988). Freshwater eel *Anguilla anguilla* (L.) from North Norway, with emphasis on occurrence, food, age and downstream migration. Nordic Journal of Freshwater Research, 64, 54–66.

[ece33099-bib-0007] Breukelaar, A. W. , Ingendahl, D. , Vriese, F. T. , de Laak, G. , Staas, S. , & Klein Breteler, J. G. P. (2009). Route choices, migration speeds and daily migration activity of European silver eels *Anguilla anguilla* in the River Rhine, northwest Europe. Journal of Fish Biology, 74, 2139–2157. 20735693 10.1111/j.1095-8649.2009.02293.x

[ece33099-bib-0008] Bruijs, M. , & Durif, C. (2009). Silver eel migration and behaviour In Van den Thillart G., Dufour S. & Rankin J. C. (Eds.), Spawning migration of the European eel. Fish and fisheries, vol. 30. (pp. 65–95). Dordrecht: Springer Science + Business Media BV.

[ece33099-bib-0009] Calles, O. , Olsson, I. C. , Comoglio, C. , Kemp, P. S. , Blunden, L. , Schmitz, M. , & Greenberg, L. A. (2010). Size‐dependent mortality of migratory silver eels at a hydropower plant, and implications for escapement to the sea. Freshwater Biology, 55, 2167–2180.

[ece33099-bib-0010] Castonguay, M. , & Durif, C. M. F. (2015). Understanding the decline in anguillid eels. ICES Journal of Marine Science, 73, 1–4.

[ece33099-bib-0011] Costa‐Dias, S. , & Lobon‐Cervia, J. (2008). Diel feeding activity and intensity in the European eel *Anguilla anguilla* (L.) during an annual cycle in a Cantabrian stream. Knowledge and Management of Aquatic Ecosystems, 390–91, 01 https://doi.org/10.1051/kmae/2008010

[ece33099-bib-0012] Crawley, M. J. (2007). The R book. Chichester, West Sussex, UK: John Wiley & Sons.

[ece33099-bib-0013] Cullen, P. , & McCarthy, T. K. (2003). Hydrometric and meteorological factors affecting the seaward migration of silver eels (*Anguilla anguilla*, L.) in the lower River Shannon. Environmental Biology of Fishes, 67, 349–357.

[ece33099-bib-0014] Dalgaard, P. (2008). Introductory statistics with R. New York, NY: Springer.

[ece33099-bib-0015] Davidsen, J. G. , Finstad, B. , Økland, F. , Thorstad, E. B. , Mo, T. A. , & Rikardsen, A. H. (2011). Early marine migration of European silver eel (*Anguilla anguilla*) in Northern Norway. Journal of Fish Biology, 78, 1390–1404. 21539549 10.1111/j.1095-8649.2011.02943.x

[ece33099-bib-0016] Deelder, C. L. (1984). Synopsis of biological data on the eel Anguilla anguilla (Linnaeus, 1758). FAO Fisheries Synopsis no. 80, revision 1: 1‐73. Food and Agriculture Organization of the United Nations

[ece33099-bib-0017] Dekker, W. (2016). Management of the eel is slipping through our hands! Distribute control and orchestrate national protection. ICES Journal of Marine Science, 73, 2442–2452.

[ece33099-bib-0018] Durif, C. , Dufour, S. , & Elie, P. (2005). The silvering process of *Anguilla anguilla*: A new classification from the yellow resident to the silver migrating stage. Journal of Fish Biology, 66, 1025–1043.

[ece33099-bib-0019] Durif, C. M. F. , & Elie, P. (2008). Predicting downstream migration of silver eels in a large river catchment based on commercial fishery data. Fisheries Management and Ecology, 15, 127–137.

[ece33099-bib-0020] Durif, C. M. F. , Gjøsæter, J. , & Vøllestad, L. A. (2011). Influence of oceanic factors on *Anguilla anguilla* (L.) over the twentieth century in coastal habitats of the Skagerrak, southern Norway. Proceedings of the Royal Society of London B: Biological Sciences, 278, 464–473. 10.1098/rspb.2010.1547 PMC3013418 20798112

[ece33099-bib-0021] Durif, C. M. , Van Ginneken, V. J. T. , Dufour, S. , Müller, T. , & Elie, P. (2009). Seasonal evolution and individual differences in silvering eels from different locations In Van den Thillart G., Dufour S. & Rankin J. C. (Eds.), Spawning migration of the European eel. Fish and fisheries, vol. 30 (pp. 13–38). Dordrechet: Springer Science + Business Media BV.

[ece33099-bib-0022] Fealy, R. , Allott, N. , Broderick, C. , de Eyto, E. , Dillane, M. , Erdil, R. M. , … White, J. (2014). RESCALE: Review and simulate climate and catchment responses at Burrishoole. Galway: Marine Institute.

[ece33099-bib-0023] Frost, W. (1950). The eel fisheries of the River Bann, Northern Ireland, and observations on the age of the silver eels. Journal du Conseil Permanent International pour l'Exploration de la Mer, 16, 358–383.

[ece33099-bib-0024] Geeraerts, C. , & Belpaire, C. (2010). The effects of contaminants in European eel: A review. Ecotoxicology, 19, 239–266. 19806452 10.1007/s10646-009-0424-0

[ece33099-bib-0025] Haraldstad, Ø. , Vøllestad, L. A. , & Jonsson, B. (1985). Descent of European silver eels, *Anguilla anguilla* L., in a Norwegian watercourse. Journal of Fish Biology, 26, 37–41.

[ece33099-bib-0026] Haro, A. (2003). Downstream migration of silver‐phase anguillid eels In Aida K., Tsukamoto K., & Yamauchi K. (Eds.), Eel biology (pp. 215–222). Tokyo: Springer‐Verlag.

[ece33099-bib-0027] Huang, Y. S. , Rousseau, K. , Le Belle, N. , Vidal, B. , Burzawa‐Gérard, E. , Marchelidon, J. , & Dufour, S. (1998). Insulin‐like growth factor‐I stimulates gonadotropin production from eel pituitary cells: A possible metabolic signal for induction of puberty. Journal of Endocrinology, 159, 43–52. 9795340 10.1677/joe.0.1590043

[ece33099-bib-0028] Huitfeldt‐Kaas, H. (1904). Aalefiskeri i ferskvand. Norsk Jæger‐ og Fisker‐Forenings Tidsskrift, 1904, 81–102 (In Norwegian).

[ece33099-bib-0029] ICES . (2016). Report of the joint EIFAAC/ICES/GFCM Working Group on Eel (WGEEL), 15 September‐22 September 2016, Cordoba, Spain. ICES CM2016/ACOM:19. 105 pp.

[ece33099-bib-0030] Jonsson, B. , & Jonsson, N. (2011). Ecology of Atlantic salmon and brown trout. Habitat as a template for life histories. Fish and Fisheries Series, 33. Dordrecht: Springer Science + Business Media BV.

[ece33099-bib-0031] Lefebvre, F. , Fazio, G. , Mounaix, B. , & Crivelli, A. J. (2013). Is the continental life of the European eel *Anguilla anguilla* affected by the parasitic invader *Anguillicoloides crassus*? Proceedings of the Royal Society B, 280, 2912–2916. 10.1098/rspb.2012.2916 PMC3574337 23325776

[ece33099-bib-0032] Lowe, R. H. (1952). The influence of light and other factors on the seaward migration of the silver eel (*Anguilla anguilla* L.). Journal of Animal Ecology, 21, 275–309.

[ece33099-bib-0033] McGrath, C. J. , O’ Leary, D. P. , Sharkey, P. J. , & Murphy, D. F. (1979). An experimental electrical guidance system for eels at Killaloe eel weir on the River Shannon In Thurow F. (Ed.), Eel research and management. Rapports et Proce`s‐Verbaux des Re′unions du Conseil International pour l'Exploration de la Mer, vol. 174 (pp. 22–31). Charlottenlund: Conseil International pour l’Exploration de la Mer.

[ece33099-bib-0034] Palm, S. , Dannewitz, J. , Prestegaard, T. , & Wickstrøm, H. (2009). Panmixia in European eel revisited: No genetic difference between maturing adults from southern and northern Europe. Heredity, 103, 82–89. 19417764 10.1038/hdy.2009.51

[ece33099-bib-0036] Poole, W. R. , Reynolds, J. D. R. , & Moriarty, C. (1990). Observations on the silver eel migrations of the Burrishoole river system, Ireland. 1959 to 1988. Internationale Revue des Gesamten Hydrobiologie, 75, 807–815.

[ece33099-bib-0037] R Core Team (2017). R version 3.3.3: A language and environment for statistical computing. Vienna, Austria: R Foundation for Statistical Computing Retrieved from https://www.R-project.org/

[ece33099-bib-0038] Reckordt, M. , Ubl, C. , Wagner, C. , Frankowski, J. , & Dorow, M. (2014). Downstream migration dynamics of female and male silver eels (*Anguilla anguilla* L.) in the regulated German lowland Warnow River. Ecology of Freshwater Fish, 23, 7–20.

[ece33099-bib-0039] Righton, D. , Westerberg, H. , Feunteun, E. , Økland, F. , Gargan, P. , Amilhat, E. , … Aarestrup, K. (2016). Empirical observations of the spawning migration of European eels: The long and dangerous road to the Sargasso Sea. Science Advances, 2, e1501694. 27713924 10.1126/sciadv.1501694 PMC5052013

[ece33099-bib-0040] Sakamoto, Y. , Ishiguro, M. , & Kitagawa, G. (1986). Akaike information criterion statistics. Dordrecht: D. Reidel Publishing Company.

[ece33099-bib-0041] Stein, F. , Doering‐Arjes, P. , Fladung, E. , Brämick, U. , Bendall, B. , & Schröder, B. (2016). Downstream migration of the European eel (*Anguilla anguilla*) in the Elbe river, Germany: Movement patterns and the potential impact of environmental factors. River Research and Applications, 32, 666–676.

[ece33099-bib-0042] Tesch, F.‐W. (2003). The eel. Oxford: Blackwell Science.

[ece33099-bib-0043] Trancart, T. , Acou, A. , De Oliveira, E. , & Feunteun, E. (2013). Forecasting animal migration using SARIMAX: An efficient means of reducing silver eel mortality caused by turbines. Endangered Species Research, 21, 181–190.

[ece33099-bib-0044] van Ginneken, V. , Durif, C. , Balm, S. P. , Boot, R. , Verstegen, M. W. A. , Antonissen, E. , & van den Thillart, G. (2007). Silvering of European eel (*Anguilla anguilla*): Seasonal changes of morphological and metabolic parameters. Animal Biology, 57, 63–77.

[ece33099-bib-0045] Vøllestad, L. A. , & Jonsson, B. (1986). Life‐history characteristics of the European eel *Anguilla anguilla* in the Imsa River, Norway. Transactions of the American Fisheries Society, 115, 864–871.

[ece33099-bib-0046] Vøllestad, L. A. , Jonsson, B. , Hvidsten, N.‐A. , & Næsje, T. F. (1994). Experimental test of environmental factors influencing the seaward migration of European silver eels. Journal of Fish Biology, 45, 641–651.

[ece33099-bib-0047] Vøllestad, L. A. , Jonsson, B. , Hvidsten, N.‐A. , Næsje, T. F. , Haraldstad, Ø. , & Ruud‐Hansen, J. (1986). Environmental factors regulating the seaward migration of European silver eels (*Anguilla anguilla*). Canadian Journal of Fisheries and Aquatic Sciences, 43, 1909–1916.

[ece33099-bib-0048] Walsh, P. J. , Foster, G. D. , & Moon, T. W. (1983). The effects of temperature on metabolism of the American eel *Anguilla rostrata* (LeSueur): Compensation in the summer and torpor in the winter. Physiological Zoology, 56, 532–540.

[ece33099-bib-0049] Westerberg, H. , & Sjöberg, N. (2014). Overwintering dormancy behaviour of the European eel (*Anguilla anguilla* L.) in a large lake. Ecology of Freshwater Fish, 24, 532–543.

